# Association between CT-derived skeletal muscle and fat indices and fracture healing following operative treatment for intertrochanteric fractures: a multicenter retrospective study

**DOI:** 10.3389/fnut.2025.1691625

**Published:** 2025-10-27

**Authors:** Enli Li, Chengbin Huang, Jiahao Tang, Yaping Jin, Yingze Zhang, Jiasen Hu

**Affiliations:** ^1^Department of Orthopedics, Affiliated Yueqing Hospital of Wenzhou Medical University, Wenzhou, China; ^2^Department of Orthopedic, The Second Affiliated Hospital and Yuying Children’s Hospital of Wenzhou Medical University, Wenzhou, China

**Keywords:** intertrochanteric fracture, skeletal muscle index, sarcopenia, computed tomography, fracture healing

## Abstract

**Background:**

Intertrochanteric fractures account for nearly 50% of hip fractures in elderly patients and are primarily treated with internal fixation. Considerable variability in postoperative healing persists, with delayed union or nonunion prolonging immobilization and increasing complications and healthcare costs. Accurate, objective assessment of healing is essential, and the Radiographic Union Score for Hip (RUSH) offers a reliable quantitative tool. Sarcopenia and visceral adiposity are linked to poor surgical outcomes, yet their roles in fracture healing remain unclear. This study hypothesizes that low skeletal muscle index (SMI) and high fat indices are associated with delayed healing.

**Methods:**

A total of 619 participants from two institutions were enrolled to assess the skeletal muscle index (SMI), subcutaneous fat index (SFI), visceral fat index (VFI), and the visceral-to-subcutaneous fat area ratio (VSR) at the T12 level, and subsequently categorized into control and experimental groups based on one-month postoperative RUSH scores (≥18 vs. <18). Fracture healing was quantified using RUSH scores assessed by six blinded orthopedists (ICC = 0.824 at 1 month). At the same follow-up, a higher RUSH score was interpreted as faster fracture healing, whereas a lower score indicated delayed healing. Slower healing was defined as a RUSH score of less than 18 at 1 month.

**Results:**

Multicenter analysis demonstrated that each unit increase in SMI was associated with a 10–29.4% reduction in the risk of slower healing (Institution 1: OR = 0.900, 95% CI: 0.859–0.942; Institution 2: OR = 0.706, 95% CI: 0.610–0.818), whereas each unit increase in VFI was associated with a 1.7–6.4% increase in risk (OR = 1.021–1.064). In both institutions, the change in RUSH score from 1 day to 1 month was positively correlated with SMI and negatively correlated with VFI, with stronger associations observed in Institution 1 (*r* = 0.591, *p* < 0.001; *r* = −0.438, *p* < 0.001). ROC analyses confirmed that SMI had better discrimination (AUC = 0.682–0.862) compared with VFI (AUC = 0.614–0.691).

**Conclusion:**

Preoperative T12-level SMI and VFI were associated with the rate of fracture healing after adjustment, with lower SMI and higher VFI linked to slower healing.

## 1 Introduction

Hip fractures represent one of the most prevalent and severe injuries in the elderly population, marked by high incidence, disability, and mortality rates, with a global disease burden that continues to increase (1). Hip fractures are generally classified, based on anatomical location, into femoral neck fractures and intertrochanteric fractures (2). Among these, intertrochanteric fractures account for approximately half of all cases and are typically managed with internal fixation (e.g., PFNA, Gamma nail, InterTAN). Postoperative healing capacity and speed have become key clinical concerns (3, 4). Although most patients achieve satisfactory healing postoperatively, significant variability in healing time remains, with some individuals developing delayed union or nonunion (5, 6). This prolongs bed rest, increases the risk of complications such as hypostatic pneumonia and deep vein thrombosis, and may necessitate revision surgery, thereby adversely impacting prognosis and quality of life. Thus, preoperative identification of individuals at high risk for delayed healing, along with the implementation of individualized interventions (e.g., nutritional support, anti-sarcopenia therapy, early weight-bearing training), is critical for optimizing clinical outcomes.

However, the assessment of fracture healing progression in clinical practice continues to rely heavily on subjective radiographic descriptions, such as “callus formation” or “fuzzy fracture line,” which are associated with considerable interobserver variability and subjectivity (7). Moreover, fracture healing is not a binary outcome but a continuous biological process. To address this issue, the Radiographic Union Score for Hip (RUSH) has been developed as a quantitative imaging assessment tool that evaluates multiple dimensions, including cortical bridging, fracture line disappearance, and trabecular consolidation (7–9). Studies have demonstrated that the RUSH score significantly improves interobserver agreement among physicians of varying specialties and experience levels when assessing intertrochanteric fractures, with the intraclass correlation coefficient (ICC) increasing from 0.50 to 0.88 (95% CI: 0.86–0.90) (7, 9). In addition, it has been shown to substantially enhance both interobserver and interobserver reliability over time. Receiver operating characteristic (ROC) analysis revealed a strong correlation between the RUSH score and actual fracture healing, with an area under the curve (AUC) of 0.989 for intertrochanteric fractures (7, 9), indicating that the RUSH score accurately reflects the status of fracture union. Therefore, the RUSH scoring system offers a more objective and dynamic method for the quantitative evaluation of postoperative healing progression.

Sarcopenia is defined as the progressive decline in skeletal muscle mass, strength, and function associated with aging, with an estimated 1% loss of muscle mass per year after the age of 40 (10, 11). The condition of sarcopenia is often accompanied by an increase in fat mass (FM) and may be worsened by obesity, known as sarcopenic obesity (SO) (12). Previous research has demonstrated a significant association between sarcopenia and higher postoperative mortality, increased risk of infection, and impaired functional recovery (13–15). This is particularly important in patients with hip fractures, as reduced skeletal muscle mass may impair perioperative resilience and hinder fracture healing potential. Fracture healing is influenced not only by bone-related factors (e.g., bone mineral density and fracture type) but also by the surrounding muscular system. Skeletal muscle is considered essential for bone repair through the secretion of myokines, mechanical stimulation, and regulation of bone formation and resorption processes (16–19).

In clinical practice, because patients are often bedridden, functional assessments of muscle—such as gait speed or handgrip strength—are frequently impractical. Additionally, dual-energy X-ray absorptiometry (DXA), which is commonly used to assess muscle mass, is not readily available in many clinical settings (10). Under such circumstances, chest computed tomography (CT) scans obtained preoperatively have been proposed as a feasible opportunistic imaging resource (20). Previous studies have indicated that muscle or adipose tissue indices at the T12 vertebral level (calculated as area/height^2^) may serve as reliable indicators of whole-body muscle and nutritional status (15, 21, 22). However, it remains unclear whether skeletal muscle and adipose tissue parameters derived from CT at the T12 level, such as the skeletal muscle index (SMI), visceral fat index (VFI), and subcutaneous fat index (SFI), can serve as predictors of postoperative fracture healing speed in patients with intertrochanteric fractures.

This study was conducted to quantify muscle and adipose tissue indices using preoperative chest CT scans and to evaluate fracture healing progression over time using postoperative RUSH scores. The objective was to assess whether these indices are independently associated with the rate of fracture healing in intertrochanteric fractures, thereby aiding preoperative risk stratification and personalized rehabilitation planning.

## 2 Methods

### 2.1 Study design and patient stratification

A multicenter retrospective study was conducted following approval from the Ethics Committees of the Second Affiliated Hospital of Wenzhou Medical University (Institution 1) and Yueqing Hospital Affiliated to Wenzhou Medical University (Institution 2). Both centers were affiliated with Wenzhou Medical University and were managed under the same standardized system, with consistent patient inclusion criteria and primary surgical approaches; follow-up and radiographic evaluations were conducted using the same RUSH scoring system. We acknowledge that minor differences may have existed in certain aspects; however, the overall process maintained a high degree of homogeneity. Preoperative and postoperative follow-up data were obtained from patients with intertrochanteric fractures who underwent intramedullary nailing between January and July 2024. A review panel composed of six orthopedic surgeons (three residents and three attending physicians, all experienced in the evaluation and management of hip fractures) independently assessed anteroposterior and lateral radiographs of the proximal femur on the affected side at postoperative day one and 1 month. Fracture healing was evaluated using the RUSH score following a blinding procedure that involved removal of imaging metadata and random presentation of the images.

The intraclass correlation coefficient (ICC) was calculated to evaluate interobserver reliability in the application of the RUSH score. A high level of agreement was observed among six orthopedic surgeons in the evaluation of postoperative day 1 radiographs (ICC = 0.774, 95% CI: 0.749–0.799), which improved to an almost perfect level for postoperative month 1 radiographs (ICC = 0.824, 95% CI: 0.803–0.844), (23). In the group analysis, attendings exhibited higher consistency in RUSH score evaluations compared with residents (attendings: postoperative day 1, ICC = 0.805, 95% CI: 0.778–0.830; postoperative month 1, ICC = 0.848, 95% CI: 0.826–0.868; residents: postoperative day 1, ICC = 0.723, 95% CI: 0.687–0.756; postoperative month 1, ICC = 0.783, 95% CI: 0.754–0.810). Final agreement on each patient’s fracture healing status was achieved through consensus among all reviewers and study investigators, based on the available imaging data.

**Figure 1:**
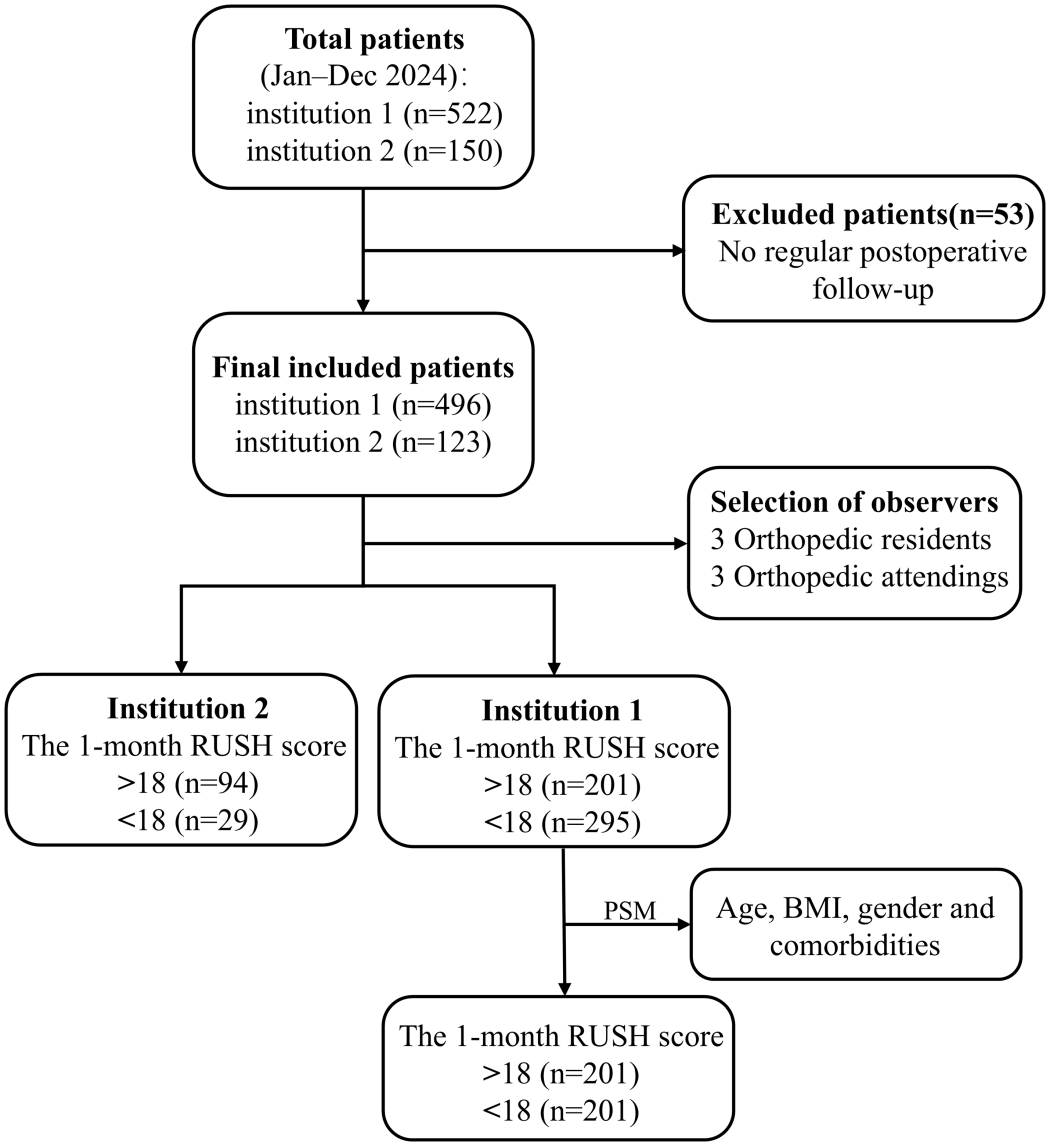
The flow chart of this study.

Based on the one-month postoperative RUSH scores, participants were divided into two groups: a control group (RUSH score≥18) and an experimental group (RUSH score < 18). Inclusion criteria were as follows: (1) a confirmed diagnosis of intertrochanteric fracture by X-ray or CT imaging, treated with intramedullary nailing; (2) a minimum interval of 30 days between two consecutive X-ray examinations for the same patient; (3) availability of complete follow-up and clinical data. Exclusion criteria included: (1) lack of chest CT examination; (2) refusal to undergo surgery; (3) presence of artifacts in CT images; (4) incomplete preoperative or postoperative follow-up data. The study methodology flowchart is presented in Figure 1. Only patients who completed preoperative chest CT were included in this study. Although preoperative chest CT was a routine evaluation for hip fracture patients at both institutions, a small number of patients were not included because they had already undergone CT at another hospital, required urgent surgery, or declined the examination for non-medical reasons. Consequently, a certain degree of selection bias may have been introduced by the inclusion criteria; however, its impact was expected to be limited.

### 2.2 RUSH scoring system for fracture healing

**Figure 2:**
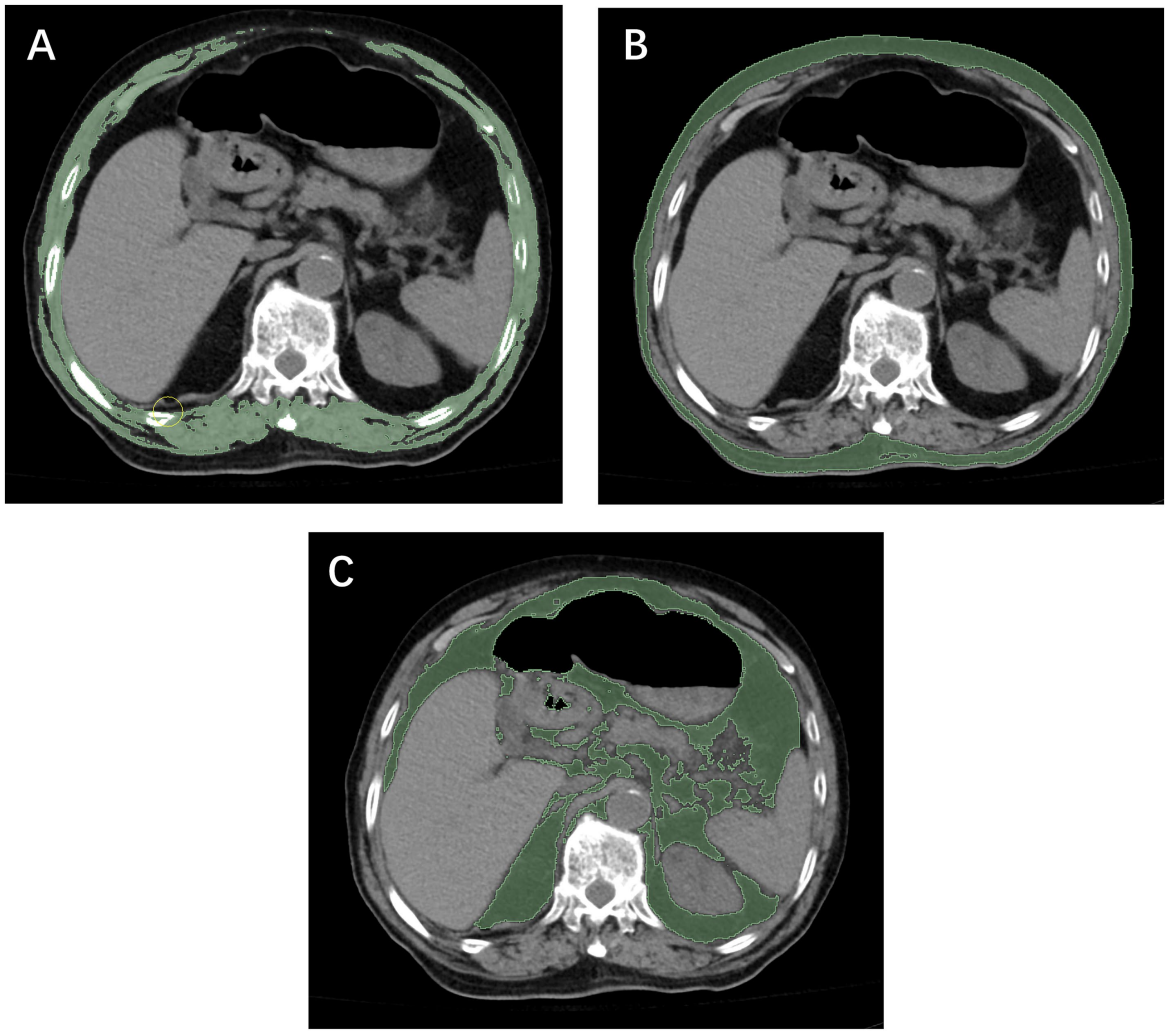
Measurement of the skeletal muscle index **(A)**, subcutaneous fat index **(B)**, and visceral fat index **(C)** using computed tomography at the T12 level.

The RUSH score sheet is completed by each reviewer, with the score based on four evaluated components: cortical bridging, cortical fracture disappearance, trabecular consolidation, and trabecular fracture disappearance. The cortical bridging index score ranges from 4 to 12 points, as each of the four cortical bones is scored individually on a scale from 1 to 3. Similarly, the cortical fracture disappearance score ranges from 4 to 12 points, as determined by the visibility of fracture lines across the four cortices. The two trabecular indices are each scored from 1 to 3, reflecting callus formation and trabecular fracture line disappearance, respectively. Therefore, the total RUSH score ranges from 10 to 30 points. As shown in the representative radiographs (Figure 2), two different patients illustrate the variability in RUSH scores applied in the evaluation of fracture healing. The postoperative day 1 radiograph is associated with a low RUSH score (12), while the 1-month postoperative radiograph demonstrates a high score (25), thereby highlighting the progression of healing as captured by the scoring system.

In the evaluation of postoperative fracture healing, the RUSH score at 1 month after surgery was selected as the primary outcome measure, with a threshold of 18 points applied to define delayed healing (RUSH <18). This cutoff was originally reported by Frank et al. for femoral neck fractures and has since been applied in studies of intertrochanteric and subtrochanteric fractures (24–29). These regions are situated within the proximal femoral stress concentration zone and share similar mechanical and biological characteristics during fracture healing. Given that stable intertrochanteric fractures generally have a comparatively rich blood supply and exhibit faster radiographic healing, setting the threshold at a later time point may lead to an overestimation of nonunion risk. Therefore, a cutoff of 18 points at 1 month postoperatively was adopted as a practical criterion for early healing, thereby providing a quantitative basis to explore the relationship between patient characteristics and early healing velocity.

### 2.3 CT-based body composition assessment

**Figure 3:**
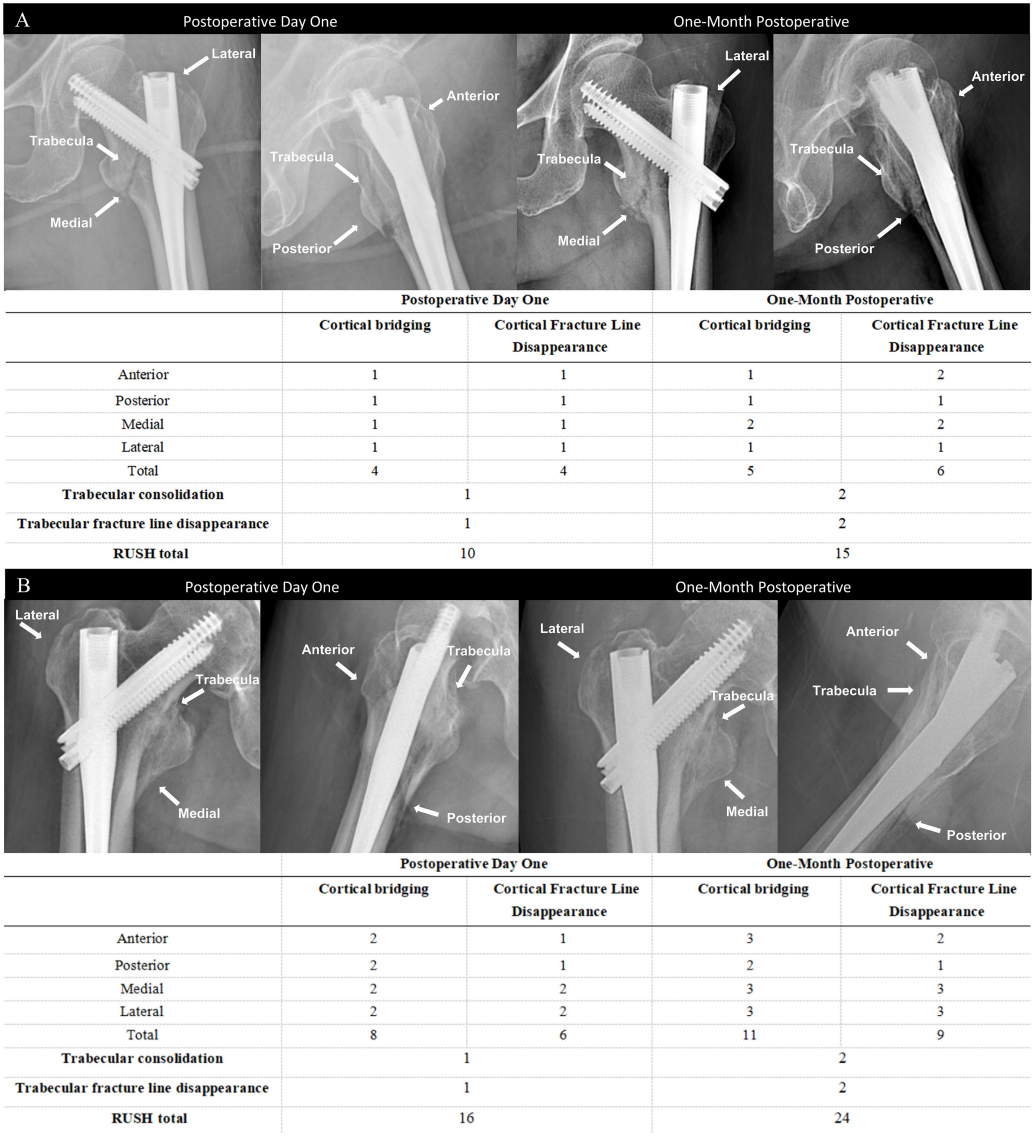
Anteroposterior and lateral radiographs obtained at postoperative day 1 and at 1 month in two patients with relatively slow **(A)** and fast **(B)** fracture healing, along with the corresponding RUSH scores.

Both institution 1 and institution 2 were affiliated with Wenzhou Medical University, and all diagnostic procedures and imaging protocols were conducted under the same standardized system. In both centers, unenhanced chest CT scans were performed using the same model of scanner (Philips Brilliance 16-slice, Philips Medical Systems, Eindhoven, the Netherlands), with identical acquisition parameters (120 kV, 250 mA, slice thickness 5 mm). All imaging data were stored in the institutional Picture Archiving and Communication System (PACS). Segmentation of skeletal muscle and adipose tissue was performed at the axial level corresponding to the mid-body level of the T12 vertebra using NIH ImageJ software (version 1.52c). Based on previously validated Hounsfield unit (HU) ranges (30, 31), skeletal muscle and adipose tissue were defined using ranges of −29 to 150 HU and −150 to −50 HU, respectively (Figure 3). The cross-sectional areas obtained were normalized to patient height by dividing by the square of the patient’s height (m^2^) to calculate the SMI, VFI, and SFI. Patients’ heights in this study were primarily obtained from hospital admission records, whereas for a small subset of patients unable to stand, measurements were taken at the bedside. Image analysis was performed by two radiological assessors, each with more than 5 years of clinical experience and proficiency in ImageJ. One assessor delineated the anatomical regions of interest, while the other independently reviewed the contours to ensure measurement accuracy.

Recent studies have demonstrated that cross-sectional muscle and adipose tissue metrics at the T12 vertebral level, derived from routine chest CT images, serve as effective markers of whole-body muscle and nutritional status (21, 22, 32). These metrics have also been associated with sarcopenia, prolonged hospital stay, short-term adverse outcomes, and 1-year mortality, and are particularly suitable for use in trauma or emergency settings due to their ready availability (33).

### 2.4 Statistical analysis

The Shapiro–Wilk test was used to evaluate the normality of data distributions. Baseline characteristics were summarized as medians with interquartile ranges (IQR), means ± standard deviations (SD), or counts with percentages (*n*,%) as appropriate. Continuous variables were compared using the Mann–Whitney *U* test or the independent *t*-test, depending on the distribution of the data. Categorical variables were analyzed using the Pearson chi-square test or Fisher’s exact test. Spearman rank correlation, conducted using R software (version 3.5.3), was employed to evaluate the association between changes in the RUSH score and skeletal muscle and fat indices (SMI, VFI, SFI). A *p*-value of <0.05 was considered statistically significant. The predictive performance of each variable for fracture healing was assessed through ROC curve analysis. All statistical analyses were performed using SPSS software (version 27.0; IBM Corp., Armonk, NY, United States).

Propensity score matching (PSM) was applied to minimize potential confounding effects in this observational study. Nine covariates were included in the matching process: age, sex, body mass index (BMI), smoking status, alcohol consumption, hypertension, diabetes mellitus, hyperlipidemia. A logistic regression model was used to generate propensity scores. Nearest neighbor 1:1 matching was then performed with a caliper of 0.05 to pair individuals between the experimental and control groups. All PSM procedures were conducted using SPSS version 27.0.

## 3 Results

### 3.1 Institution 1

After rigorous screening, a total of 496 patients were included from Institution 1. Based on their RUSH scores at 1 month postoperatively, patients were divided into two groups: 295 individuals with scores below 18 and 201 with scores above 18. No significant baseline differences were noted between the groups. To reduce the influence of confounding factors and selection bias, propensity score matching (PSM) was conducted using age, sex, BMI, affected limb, and existing comorbidities as covariates. Matching was performed in a 1:1 ratio with a caliper width of 0.05, yielding 201 matched pairs with balanced characteristics. Post-matching comparisons showed no significant differences in age, sex, BMI, smoking, alcohol consumption, or comorbidities between the groups (Table 1).

**Table 1:** Clinical baseline characteristics of the raw cohort samples and propensity matched participants at institutions 1.

Variable	Institution 1			Propensity-matched Institution 1		
	RUSH < 18 group (*n* = 295)	RUSH > 18 group (*n* = 201)	*p*-value	RUSH < 18 group (*n* = 201)	RUSH > 18 group (*n* = 201)	*P*-value
Age, (years)	81 (72–88)	82 (68.5–86.5)	0.350	82 (73–88)	82 (68.5–86.5)	0.130
BMI, (kg/m^2^)	22.01 ± 3.16	21.82 ± 3.41	0.523	22.10 ± 3.15	21.82 ± 3.41	0.407
Gender, *n*(%)			0.726			0.915
Female	204 (69.2)	136 (67.7)		137 (68.2)	136 (67.7)	
Male	91 (30.8)	65 (32.3)		64 (31.8)	65 (32.2)	
Injured limb, *n*(%)			0.465			0.599
Left	170 (57.6)	113 (56.2)		115 (57.2)	113 (56.2)	
Right	125 (42.4)	88 (43.8)		86 (42.8)	88 (43.8)	
Current smoking, n(%)	2 (0.7)	0 (0)	0.242	0 (0)	0 (0)	–
Current drinking, *n*(%)	1 (0.3)	0 (0)	0.409	0 (0)	0 (0)	–
Hypertension, *n*(%)	156 (52.9)	98 (48.8)	0.367	101 (50.2)	98 (48.8)	0.765
Diabetes, *n*(%)	71 (24.1)	38 (18.9)	0.173	45 (22.4)	38 (18.9)	0.388
Hyperlipidemia, *n*(%)	4 (1.4)	4 (2.0)	0.582	3 (1.5)	4 (2.0)	0.703

BMI, body mass index.

Using ImageJ software, skeletal muscle and fat indices were compared between the two groups (Table 2). Before matching, the experimental group exhibited a lower SMI and a higher VFI than the control group (31.80 vs. 35.99, *p* < 0.001; 40.44 vs. 33.98, *p* < 0.001), whereas SFI, ASA score, fracture type, injury mechanism, and type of internal fixation demonstrated no significant differences. After matching, SMI and VFI differed significantly between the two groups (32.10 vs. 36.18, *p* < 0.001; 40.89 vs. 33.98, *p* < 0.001), with no significant differences observed in other indicators.

**Table 2:** The skeletal muscle indices, adipose indices, preoperative factors, and intraoperative characteristics of the raw cohort samples and propensity score-matched participants at Institution 1.

Variable	Institution 1			Propensity-matched Institution 1		
	RUSH < 18 group (*n* = 295)	RUSH > 18 group (*n* = 201)	*P*-value	RUSH < 18 group (*n* = 201)	RUSH > 18 group (*n* = 201)	*P*-value
SMI, (cm^2^/m^2^)	31.80 ± 6.20	35.99 ± 6.00	<0.001	32.10 ± 6.33	36.18 ± 6.00	<0.001
VFI, (cm^2^/m^2^)	40.44 (31.10–49.82)	33.98 (24.36–49.12)	<0.001	40.89 (31.24–50.47)	33.98 (24.36–49.12)	<0.001
SFI, (cm^2^/m^2^)	28.40 (21.22–38.40)	25.95 (20.17–43.60)	0.420	28.69 (21.16–39.19)	25.95 (20.17–43.60)	0.444
VSR	1.37 (0.98–1.93)	1.18 (0.76–1.85)	0.020	1.36 (0.98–1.86)	1.21 (0.79–1.88)	0.098
ASA score, *n*(%)			0.338			
I or II	216 (73.3)	133 (66.2)		142 (70.7)	133 (66.2)	0.622
III or above	79 (26.8)	68 (33.8)		59 (29.4)	68 (33.8)	
Fracture type, *n*(%)						1
Stable	289 (98.0)	198 (98.5)		199 (99.0)	199 (99.0)	
Reverse	6 (2.0)	3 (1.5)		2 (1.0)	2 (1.0)	
Injury mechanism, *n*(%)			0.436			1
Low-energy trauma	272 (92.2)	189 (94.0)		189 (94.0)	189 (94.0)	
High-energy trauma	23 (7.8)	12 (6.0)		2 (6.0)	2 (6.0)	
Type of internal fixation, *n*(%)			0.603			0.302
PFNA	60 (20.3)	47 (23.4)		37 (18.4)	47 (23.4)	
Gamma nail	3 (1.0)	1 (0.5)		3 (1.5)	1 (0.5)	
InterTAN nail	232 (78.6)	153 (76.1)		161 (80.1)	153 (76.1)	

SMI, Skeletal muscle index; ASA, American Society of Anesthesiologists; PFNA, Proximal Femoral Nail Autorotation. InterTAN, Intertrochanteric Antegrade Nail; VSR, Visceral-to-subcutaneous ratio of fat area; Gamma nail, Gamma Intramedullary Nail; VFI, Visceral fat index; SFI, Subcutaneous fat index.

Conditional logistic regression models were constructed using Age, SMI, VFI, and the visceral-to-subcutaneous fat area ratio (VSR) (Table 3). The results indicated that SMI (OR = 0.900, 95%CI: 0.859–0.942, *p* < 0.001) was independently identified as a protective factor, while higher VFI (OR = 1.017, 95% CI: 1.001–1.034, *p* = 0.036) was significantly associated with elevated risk. Age and VSR were not independently associated with the outcome (*p* > 0.05). Each 1-unit increase in SMI was associated with a nearly 10% reduction in the risk of slower fracture healing, whereas each 1-unit increment in VFI was associated with a statistically significant 1.7% increase in risk. A greater muscle mass was associated with more rapid fracture healing, whereas increased visceral fat accumulation was associated with delayed healing.

**Table 3:** Conditional logistic regression analysis of selected skeletal muscle indices and adipose indices (Institution 1).

Influence factors	*B*	S. E	Ward	OR	95%CI	*P*
Age, (years)	−0.006	0.011	0.297	0.994	0.972–1.016	0.585
SMI,(cm2/m2)	−0.106	0.023	20.477	0.900	0.859–0.942	<0.001
VFI,(cm2/m2)	0.017	0.008	4.386	1.017	1.001–1.034	0.036
VSR	−0.263	0.160	2.700	0.768	0.561–1.052	0.100

SMI, Skeletal muscle index; VFI, Visceral fat index; SFI, Subcutaneous fat index; VSR, Visceral-to-subcutaneous ratio of fat area.

### 3.2 Institution 2

A total of 123 patients were enrolled at Institution 2. Based on their one-month postoperative RUSH scores, patients were divided into two groups: 29 with scores below 18 and 94 with scores above 18. Statistically significant differences were observed between the two groups in terms of SMI, VFI, VSR, and type of internal fixation (all *p* < 0.05). However, no significant differences were found in BMI, age, SFI, injured limb, comorbidities, gender, ASA score, fracture type, injury mechanism, smoking status, or alcohol consumption. Detailed results are presented in Table 4.

**Table 4:** Clinical baseline characteristics, skeletal muscle indices, adipose indices, preoperative factors and intraoperative characteristics of the participants at institutions 2.

Variable	Institution 2		
	RUSH < 18 group (*n* = 29)	RUSH > 18 group (*n* = 94)	*P*-value
Age, (years)	83 (70–88)	75.5 (63.75–86)	0.183
BMI, (kg/m^2^)	22.22 (21.28–23.88)	22.03 (20.41–24.68)	0.638
Gender, *n*(%)			0.521
Female	18 (62.1)	52 (55.3)	
Male	11 (37.9)	42 (44.7)	
Injured limb, *n*(%)			0.673
Left	17 (58.6)	51 (54.3)	
Right	12 (41.4)	43 (45.7)	
Current smoking, *n*(%)	4 (13.8)	22 (23.4)	0.268
Current drinking, *n*(%)	4 (13.8)	19 (20.2)	0.438
Hypertension, *n*(%)	14 (48.3)	46 (48.9)	0.950
Diabetes, *n*(%)	11 (37.9)	23 (24.5)	0.156
Hyperlipidemia, *n*(%)	3 (10.3)	13 (13.8)	0.626
SMI, (cm^2^/m^2^)	25.30 (22.38–29.35)	34.51 (29.57–37.92)	<0.001
VFI, (cm^2^/m^2^)	37.88 (29.61–60.75)	29.36 (22.22–36.21)	0.002
SFI, (cm^2^/m^2^)	25.48 (18.18–44.25)	30.36 (18.73–43.06)	0.575
VSR	1.39 (1.17–1.78)	1.11 (0.68–1.47)	0.001
ASA score, *n*(%)			0.663
I or II	23 (79.3)	66 (70.3)	
III or above	6 (20.7)	28 (29.8)	
Fracture type, *n*(%)			0.847
Stable	28 (96.6)	90 (95.7)	
Reverse	1 (3.4)	4 (4.3)	
Injury mechanism, *n*(%)			0.273
Low-energy trauma	23 (79.3)	81 (86.2)	
High-energy trauma	6 (20.7)	13 (13.8)	
Type of internal fixation, *n*(%)			0.023
PFNA	6 (20.7)	6 (6.4)	
Gamma nail	0 (0)	0()	
InterTAN nail	23 (79.3)	88 (93.6)	

BMI, body mass index; SMI, Skeletal muscle index; ASA, American Society of Anesthesiologists; PFNA, Proximal Femoral Nail Autorotation; InterTAN, Intertrochanteric Antegrade Nail; Gamma nail, Gamma Intramedullary Nail; VFI, Visceral fat index; SFI, Subcutaneous fat index; VSR, Visceral-to-subcutaneous ratio of fat area.

Age, SMI, VSR, VFI, and type of internal fixation were included in the univariate analysis (Table 5). Variables with a *p*-value less than 0.2-namely SMI, VSR, VFI, and type of internal fixation—were subsequently included in the multivariate logistic regression analysis. The results indicated that SMI (OR = 0.706, 95% CI: 0.610–0.818, *p* < 0.001) and VFI (OR = 1.064, 95% CI: 1.019–1.111, *p* = 0.018) were identified as independent predictors of slower fracture healing. Each 1-unit increase in SMI was associated with a nearly 29.4% reduction in slower fracture healing risk, whereas each 1-unit increment in VFI corresponded to a statistically significant 6.4% elevation in risk. Similarly, a higher muscle mass was consistently associated with more rapid fracture healing, whereas increased visceral fat accumulation was associated with an increased likelihood of impaired healing.

**Table 5:** Univariate and multivariate logistic regression analysis of risk factors (Institution 2).

Influence factors	Univariable			Multivariable		
	OR	95%CI	*P*	OR	95%CI	*P*
Age, (years)	1.019	0.988–1.051	0.233			
SMI, (cm^2^/m^2^)	0.774	0.694–0.863	<0.001	0.706	0.610–0.818	<0.001
VFI, (cm^2^/m^2^)	1.039	1.014–1.066	0.002	1.064	1.019–1.111	0.018
VSR	2.417	1.278–4.571	0.007	1.277	0.568–2.867	0.148
Type of internal fixation, *n*(%)	0.511	0.278–0.941	0.031	0.69	0.311–1.537	0.263

SMI, Skeletal muscle index; SFI, Subcutaneous fat index; VSR, Visceral-to-subcutaneous ratio of fat area; VFI, Visceral fat index.

### 3.3 Correlation of change in RUSH score (1 m-1 d) with SMI and VFI

**Figure 4:**
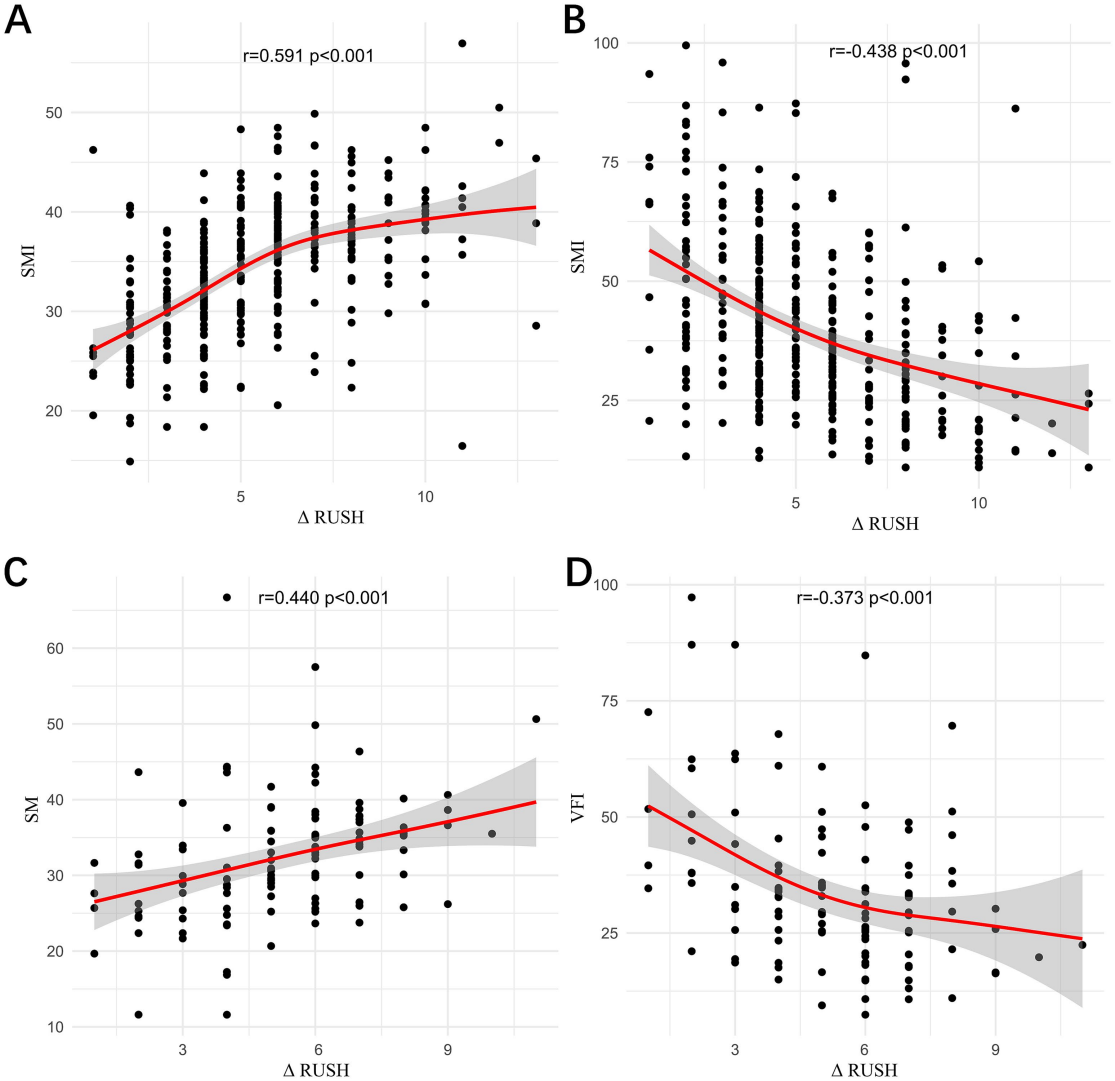
The correlations between the change in RUSH score (1 m–1 d) and SMI in Institution 1 **(A)**, between the change in RUSH score (1 m–1 d) and VFI in Institution 1 **(B)**, between the change in RUSH score (1 m–1 d) and SMI in Institution 2 **(C)**, and between the change in RUSH score (1 m–1 d) and VFI in Institution 2 **(D)** were evaluated.

In Institution 1 (Figure 4), a moderate positive correlation was observed between the change in RUSH score (1 month-1 day) and SMI (*r* = 0.591, *p* < 0.001), while a moderate negative correlation was noted with VFI (*r* = −438, *p* < 0.001). In Institution 2, a moderate positive correlation was observed between the change in RUSH score (1 m-1 d) and SMI (*r* = 0.440, *p* < 0.001), and a weak negative correlation was noted with VFI (*r* = −0.373, *p* < 0.001).

Across both institutions, SMI was found to have a positive correlation with the change in RUSH score, whereas VFI was found to be negatively correlated with the change in RUSH score, with a stronger association observed in Institution 1 compared to Institution 2. This inconsistency may be attributable to the smaller sample size in Institution 2, underscoring the need for further analyses with a larger cohort. Therefore, preoperative muscle mass and visceral fat content may serve as reliable predictors of postoperative fracture healing.

### 3.4 Receiver operator characteristics analysis

**Figure 5:**
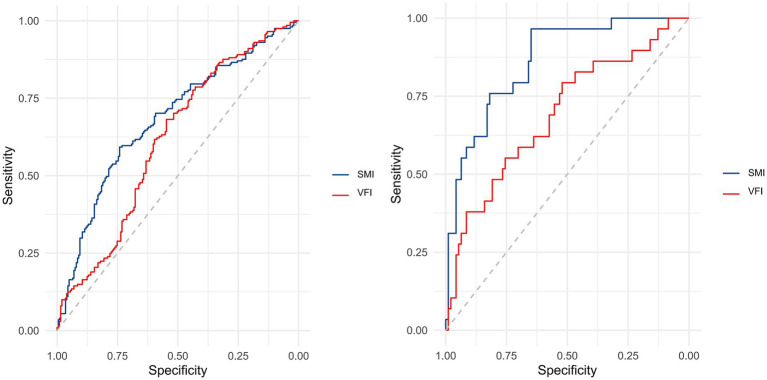
Receiver operating characteristic (ROC) analysis was performed to assess the discriminative capacity of SMI and VFI for identifying patients with high versus low fracture-healing potential at 1-month postoperative follow-up. In Institution 1, ROC curves for both SMI and VFI were generated (left panel), while corresponding curves for Institution 2 are presented in the right panel.

The results of ROC curve analyses, as shown in Supplementary Tables S1, S2, were used to assess the discriminative ability of SMI and VFI for identifying patients with high versus low fracture-healing potential at 1 month postoperatively. Using a postoperative 1-month RUSH score greater than 18 as the state variable, ROC curves were generated separately for Institution 1 (SMI and VFI) and Institution 2 (SMI and VFI) (Figure 5). In Institution 1, the ROC analysis yielded the following area under the curve (AUC) values: SMI, 0.682 (95% CI: 0.629–0.734, *p* < 0.001; significant); VFI, 0.614 (95%CI: 0.559–0.669, *p* < 0.001; significant). For Institution 2, the AUC values were: SMI, 0.862 (95% CI: 0.790–0.933, *p* < 0.001; significant); VFI, 0.691 (95% CI: 0.577–0.806, *p* = 0.001; significant).

For Institution 1, the optimal cutoff values, as determined by the Youden index, were 33.046 cm^2^/m^2^ for SMI and 35.270 cm^2^/m^2^ for VFI. At these thresholds, the PPV and NPV for SMI were 30.4% (95% CI: 0.235–0.373) and 35.5% (95% CI: 0.293–0.417), respectively; for VFI, they were 63.2% (95% CI: 0.561–0.704) and 60.1% (95% CI: 0.537–0.664), respectively. For Institution 2, the optimal cutoff values, as determined by the Youden index, were 31.841 cm^2^/m^2^ for SMI and 29.526 cm^2^/m^2^ for VFI. At these thresholds, the PPV and NPV for SMI were 54.1% (95% CI: 0.416–0.666) and 1.6% (95% CI: <0.001–0.047), respectively; for VFI, they were 89.1% (95% CI: 0.809–0.973) and 33.8% (95% CI: 0.226–0.451), respectively.

## 4 Discussion

This study measured muscle and fat indices at the T12 vertebral level using preoperative chest CT images and, for the first time, examined their correlation with postoperative RUSH scores to assess their predictive value for the fracture healing rate in patients with intertrochanteric femoral fractures. The results revealed that patients with lower SMI and higher VFI were at an increased risk of delayed healing. After adjusting for confounding factors such as age, BMI, and comorbidities, both indices were independently associated with slower fracture healing. These findings suggest that low muscle mass and excessive visceral fat are strongly associated with postoperative slower fracture-healing, supporting the use of SMI and VFI as important indicators for preoperative risk stratification.

The threshold of RUSH ≥18 at 1 month postoperatively was adopted as a reference indicator of substantial radiographic healing, primarily to evaluate early healing progression rather than to strictly define eventual nonunion. Previous studies have demonstrated a significant negative correlation between the RUSH score at approximately 1 month postoperatively and VAS pain scores in intertrochanteric fracture patients, whereas this correlation was no longer significant at 90 days (26). This finding suggests that the 1-month RUSH score is a more sensitive indicator of early recovery, thereby supporting the clinical validity of the 18-point cutoff. In addition, the 1-month postoperative period represents both the critical stage of primary callus formation and a routine clinical follow-up timepoint. Healing status at this stage often determines whether patients can initiate weight-bearing and serves as a crucial window for identifying potential healing impairments. Therefore, a clearly defined threshold facilitates the timely identification of patients with delayed healing in clinical practice, enabling adjustment of rehabilitation and anti-osteoporosis strategies. Although this cutoff was originally derived from femoral neck fracture studies, its application in intertrochanteric and subtrochanteric fractures has been supported to some extent. The exploratory nature of this cutoff is emphasized in the present study, and it is acknowledged that while the threshold has reference value, it does not constitute a definitive standard for diagnosing nonunion or predicting long-term outcomes. Further validation through large-scale and site-specific studies is warranted.

Sarcopenia is an age-related syndrome characterized by progressive decline in skeletal muscle mass and muscle function, and is strongly associated with frailty, falls, physical disability, reduced quality of life, and increased mortality risk (18, 34). Studies have shown that muscle mass progressively declines with age, and the prevalence of sarcopenia among patients aged 65 years and older with hip fractures is as high as 37% (10), indicating a significant clinical burden within this population.

Extensive evidence has indicated that sarcopenia constitutes not only a significant risk factor for hip fractures but is also closely linked to adverse postoperative outcomes, including refracture, infection, increased complications, and elevated mortality rates (35–37). It has been demonstrated that skeletal muscle contributes to bone repair through multiple mechanisms. Mechanical loading is induced by muscle contraction is known to regulate the mechanical microenvironment at the fracture site, thereby influencing the formation, differentiation, and mineralization of callus tissue (18, 19). Additionally, the dynamic balance of local inflammatory responses is maintained by muscle tissue through modulation of macrophage phenotypes, such as promotion of M2 macrophage recruitment, thereby creating a favorable microenvironment for fracture repair (38). Furthermore, the regulation of osteogenesis and bone remodeling is facilitated by muscle through the secretion of myogenic factors, including IGF-1, FGF-23, myostatin, and muscle-derived stromal cells (MDSCs) (16–18, 39). Therefore, reductions in both skeletal muscle quantity and quality may result in disruption of the postoperative local biomechanical and immune microenvironment, reduced weight-bearing tolerance, inhibition of callus formation, and ultimately, delayed fracture healing.

Previous studies have indicated that excessive accumulation of visceral adipose tissue (VAT) may impair skeletal metabolic homeostasis through multiple pathways (40–43). The potential mechanisms are as follows: First, VAT is known to secrete various pro-inflammatory cytokines, such as IL-1β, IL-6, and TNF-α, thereby activating the RANKL/RANK/OPG signaling axis, which promotes osteoclast differentiation and suppresses osteoblastic activity. Second, adipokines derived from VAT, such as leptin, although capable of stimulating osteoblast differentiation, may indirectly suppress bone formation by activating the sympathetic nervous system. Adiponectin has been reported to stimulate osteoblast proliferation via the MAPK signaling pathway; however, it may concurrently enhance osteoclast genesis by upregulating RANKL and inhibiting the secretion of osteoprotegerin. Additionally, VAT accumulation is capable of inducing insulin resistance, subsequently leading to reduced levels of growth hormone and insulin-like growth factor-1 (IGF-1) two essential hormones for maintaining bone homeostasis and regulating lipid metabolism. The reduction of these hormones may further compromise osteogenesis and worsen impairments in bone repair. Taken together, the pro-inflammatory microenvironment induced by VAT, dysregulation of adipokines, and suppression of the IGF-1 signaling pathway may collectively disrupt the dynamic equilibrium between bone formation and resorption, thereby leading to slower fracture healing.

Recently, growing attention has been directed toward body composition, particularly due to its impact on clinical outcomes. The clinical significance of this study lies in the fact that opportunistic chest CT images can be obtained preoperatively and utilized as an early predictive tool to identify high-risk individuals, thereby facilitating the development of individualized rehabilitation strategies. For patients at high risk of sarcopenia, preoperative nutritional interventions, including protein or branched-chain amino acid supplementation, should be implemented, and postoperative weight-bearing regimens should be adjusted accordingly (e.g., delayed or reduced loading). For patients with excessive visceral fat, preoperative optimization of glycemic and lipid control is recommended to mitigate the adverse effects of inflammation on fracture healing, thereby promoting a shift from reactive to proactive management.

This study provides valuable preliminary evidence regarding the association between SMI, VFI, and fracture healing in intertrochanteric fractures; however, several limitations should be acknowledged. First, the retrospective design inherently limits the ability to infer causality, and the relatively small sample size, along with the absence of an *a priori* power calculation, may restrict the generalizability of the findings. Second, although efforts were made to control for major covariates, several important confounding factors were not incorporated into the analysis—such as reduction quality (e.g., tip–apex distance), implant type and surgeon-related factors, time to surgery, postoperative weight-bearing protocol, osteoporosis management, vitamin D supplementation, steroid use, diabetes control, and nutritional status—due to the limitations of the available dataset. Third, inter- and intra-rater reliability for muscle and fat ROI segmentation and intraobserver ICC for RUSH scoring were not assessed, which may affect measurement consistency. Finally, since the findings were derived from CT-based body composition measurements, their applicability to clinical settings in which chest CT is not routinely conducted may be limited. In these contexts, alternative techniques for evaluating muscle mass—such as bioelectrical impedance analysis (BIA), dual-energy X-ray absorptiometry (DXA), ultrasound, or simple clinical assessments (e.g., grip strength or calf circumference)—may be necessary to approximate the corresponding measurements. Future prospective multicenter studies with larger cohorts, multimodal imaging modalities (CT/MRI), and integrated functional assessments are warranted to validate and extend these findings.

## 5 Conclusion

In conclusion, preoperative T12-level SMI and VFI were associated with the rate of fracture healing after adjustment, with lower SMI and higher VFI linked to slower healing.

## Data Availability

The original contributions presented in the study are included in the article/Supplementary material, further inquiries can be directed to the corresponding authors.

## Supplementary material

SUPPLEMENTARY FIGURE S1 Flow diagram of patient enrollment and exclusion in the two institutions.

## Glossary

BMI: Body mass index
SMI: Skeletal muscle index
VFI: Visceral fat index
SFI: Subcutaneous fat index
ASA: American Society of Anesthesiologists
PFNA: Proximal femoral nail anti-rotating
DXA: Dual energy x-ray absorptiometry
CT: Computed tomography
ROC: Receiver operating characteristic
PSM: Propensity score matching
RUSH: Radiographic Union Score for Hip
ICC: Intraclass correlation coefficient
AUC: Area under the curve
PACS: Picture Archiving and Communication System
HU: Hounsfield unit
IQR: Interquartile ranges
SD: Standard deviations
MDSCs: Muscle-derived stromal cells
SMD: Skeletal muscle density
FM: Fat mass
SO: Sarcopenic obesity
VAT: Visceral adipose tissue
VSR: Visceral-to-subcutaneous fat area ratio
