# Supplementary figures and images for: Association between CT-derived skeletal muscle and fat indices and fracture healing following operative treatment for intertrochanteric fractures: a multicenter retrospective study
Source: Front Nutr. 2025 Oct 27;12:1691625. doi: 10.3389/fnut.2025.1691625 (PMC12597792; doi:10.3389/fnut.2025.1691625)

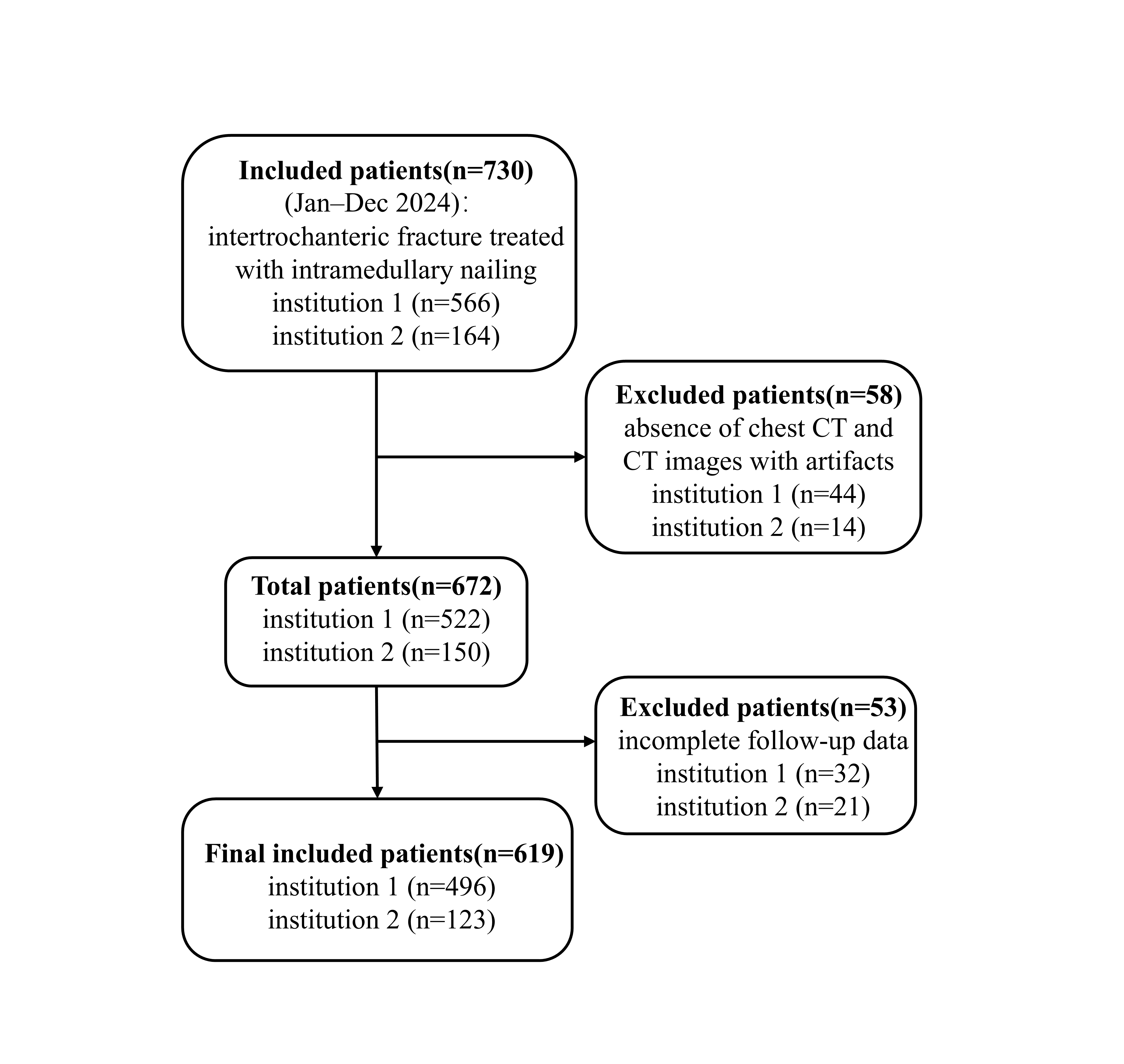

Supplement: SUPPLEMENTARY FIGURE S1 — Flow diagram of patient enrollment and exclusion in the two institutions. [file Image_1.tif]
