# Supplementary material for: Association between CT-derived skeletal muscle and fat indices and fracture healing following operative treatment for intertrochanteric fractures: a multicenter retrospective study
Source: Front Nutr. 2025 Oct 27;12:1691625. doi: 10.3389/fnut.2025.1691625 (PMC12597792; doi:10.3389/fnut.2025.1691625)
Supplement: Supplementary file 2 [file Table_1.docx]

**Table S1.** Receiver operator characteristics analysis of SMI and VFI (Institution 1).

| Influence factors | AUC (95%CI) P PPV (95%CI) NPV (95%CI) Cut off | | | | |
| --- | --- | --- | --- | --- | --- |
| SMI | 0.682 (0.629-0.734) | <0.001 | 0.304 (0.235-0.373) | 0.355 (0.293-0.417) | 33.046 |
| VFI | 0.614 (0.559-0.669) | <0.001 | 0.632 (0.561-0.704) | 0.601 (0.537-0.664) | 35.270 |

Abbreviation, SMI, Skeletal muscle index; VFI, Visceral fat index; VSR, Visceral-to-subcutaneous ratio of fat area; PPV, Positive predictive value; NPV, Negative predictive value.
