# Supplementary material for: Association between CT-derived skeletal muscle and fat indices and fracture healing following operative treatment for intertrochanteric fractures: a multicenter retrospective study
Source: Front Nutr. 2025 Oct 27;12:1691625. doi: 10.3389/fnut.2025.1691625 (PMC12597792; doi:10.3389/fnut.2025.1691625)
Supplement: Supplementary file 3 [file Table_2.docx]

**Table S2.** Receiver operator characteristics analysis of SMI and VFI (Institution 2).

| Influence factors | AUC (95%CI) P PPV (95%CI) NPV (95%CI) Cut off | | | | |
| --- | --- | --- | --- | --- | --- |
| SMI | 0.862 (0.790-0.933) | <0.001 | 0.541(0.416-0.666) | 0.016(<0.001-0.047) | 31.841 |
| VFI | 0.691(0.577-0.806) | 0.001 | 0.891(0.809-0.973) | 0.338(0.226-0.451) | 29.526 |

Abbreviation, SMI, Skeletal muscle index; PPV, Positive predictive value; NPV, Negative predictive value.
